# Short-term outcomes of pediatric HeartMate 3 left ventricular assist device: single center experience

**DOI:** 10.1093/eschf/xvaf017

**Published:** 2026-01-08

**Authors:** Mohannad Dawary, Dimpna Albert-Brotons, Felix W Tsai

**Affiliations:** King Faisal Specialist Hospital & Research Center, Heart Center, Cardiac Surgery, MBC#16, P O BOX 3354, Riyadh 11211, Saudi Arabia; King Faisal Specialist Hospital & Research Center, Heart Center, Pediatric Cardiology, Riyadh 11211, Saudi Arabia; King Faisal Specialist Hospital & Research Center, Heart Center, Cardiac Surgery, MBC#16, P O BOX 3354, Riyadh 11211, Saudi Arabia

**Keywords:** Pediatric heart failure, Heart transplantation, Ventricular assist device

## Abstract

**Introduction:**

Heart failure in paediatric patients is a complex and challenging condition. While heart transplantation is the gold standard for treating end-stage heart failure, limited donor availability necessitates alternative therapies, such as ventricular assist devices (VADs). The HeartMate 3 (HM3) VAD use in adults is well-established, but there is less data regarding outcomes in children. This study aims to evaluate the short-term outcomes of the first paediatric HM3 VAD programme in Saudi Arabia.

**Methods:**

We conducted a retrospective cohort study involving 15 consecutive paediatric patients treated with HM3 at a single tertiary cardiac centre from 2022 to 2024. Postoperative outcomes included stroke, device thrombosis, driveline infection, hospital mortality, follow-up survival and time to transplantation.

**Results:**

The cohort had a mean age of 11.07 years, with a predominance of females (67%). Average body surface area was 1.2 m^2^ (range 0.85–1.76 m^2^). The most frequent diagnosis was dilated cardiomyopathy (*n* = 13, 87%). The median lengths of ICU and hospital stays were 42 days and 56 days, respectively. The complications included strokes (*n* = 2, 13%), re-exploration for bleeding (*n* = 1, 7%), driveline infection (*n* = 2, 14%) and device thrombosis (*n* = 1, 7%). In-hospital mortality occurred in two patients. The median follow-up period was 17 months, and 60% (*n* = 9) of patients underwent successful transplantation. No mortality was reported beyond hospital discharge, and the overall 1-year survival was 87%. The 1-year transplantation-free survival rate was 55%.

**Conclusion:**

HM3 LVADs have favourable safety profiles and survival outcomes in paediatric patients with end-stage heart failure. These findings support HM3’s as a viable option for managing advanced heart failure in the Saudi paediatric population, with further studies warranted to assess long-term outcomes.

## Graphical Abstract

**Graphical Abstract:**
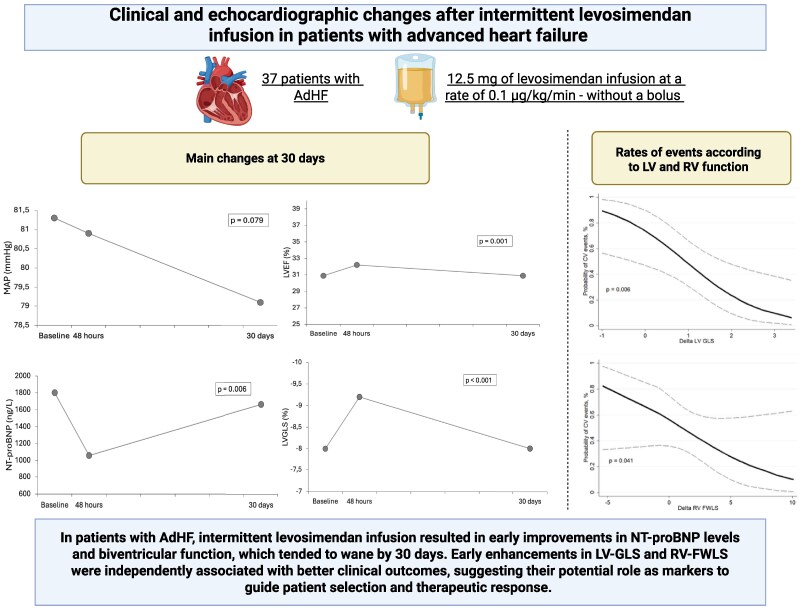
Pediatric patients with advanced heart failure were supported using the HeartMate 3 left ventricular assist device. This study summarizes patient characteristics, perioperative course, and short-term outcomes, demonstrating the feasibility of HeartMate 3 use in selected pediatric patients.

## Introduction

Heart failure in children is a complex and heterogeneous condition that poses a significant risk of morbidity and mortality. Diagnosing heart failure within the paediatric population presents considerable challenges, and research in this area is relatively scarce, as most studies focus on adult patients.^1,2^ Moreover, the aetiologies and pathophysiological mechanisms of heart failure in children differ substantially from those in adults. Congenital heart defects are the leading cause of heart failure in this population whereas ischaemic heart disease is the most common cause in the adult population.^1,3,4^ Typically, congenital malformations lead to high-output cardiac failure, whereas cardiomyopathy is associated with low-output failure. Heart failure is linked to ∼20% of congenital heart disease cases, and the incidence of heart failure following early surgical intervention is estimated at 10%^1,5^

There is no official report on the incidence of cardiomyopathy in the paediatric population. Worldwide, the incidence of dilated cardiomyopathy is reported to be 0.57–1.13 cases per 100 000 individuals, and restrictive cardiomyopathy is associated with up to 5% of all cases of cardiomyopathies in this population.^6,7^ In Saudi Arabia, most referrals for heart transplantation are due to familial dilated cardiomyopathy.

Heart transplantation remains the optimal long-term solution for end-stage heart failure. However, Saudi Arabia only performs about 40 heart transplants annually, underscoring a substantial gap in meeting the needs for heart transplantation in the region. Factors such as donor availability, size matching, cultural acceptance, and lengthy waiting lists pose significant limitations^8,9^ in Saudi Arabia. This is in contrast with the USA, where the incidence of heart transplants per million population is 100-fold the rate of heart transplant in Saudi Arabia.^10,11^

Consequently, ventricular assist devices (VADs) have emerged as a groundbreaking alternative for managing advanced heart failure in paediatric patients, serving as a bridge to transplantation and increasing survival rates.^12^

The use of the HM3 (Abbott, Chicago, IL, USA) VAD in paediatric patients represents a new era of mechanical circulatory support. Data regarding its use in the paediatric population remain limited, and few studies have investigated the short- and long-term outcomes of this device.^12^ A multicenter registry analysis of HM3 use in patients with congenital heart disease indicated a low incidence of adverse events and mortality, reporting only one death among Fontan patients.^13^ The Advanced Cardiac Therapies Improving Outcomes Network (ACTION) study revealed that device-related adverse events were more prevalent in patients with a low body surface area (BSA).^14^ Lastly, data from the MOMENTUM 3 trial, which included 1028 adult patients, demonstrated that the HM3 device has a lower incidence of ischaemic and haemorrhagic strokes than the HM2 device.^15^

As the HM3 device is newly introduced in paediatric patients in Saudia Arabia, This programme is built on a multidisciplinary team comprising cardiac surgeons, heart failure cardiologists, ICU staff, perfusionists, and outpatient clinic educators to ensure comprehensive patient follow-up after discharge. Comprehensive analyses of device-related events, such as stroke, driveline infections, and thromboembolic events, will need to be monitored and compared with international standards. Thus, this study aimed to describe the short-term outcomes associated with the HM3 device in paediatric patients.

**Figure 1:**
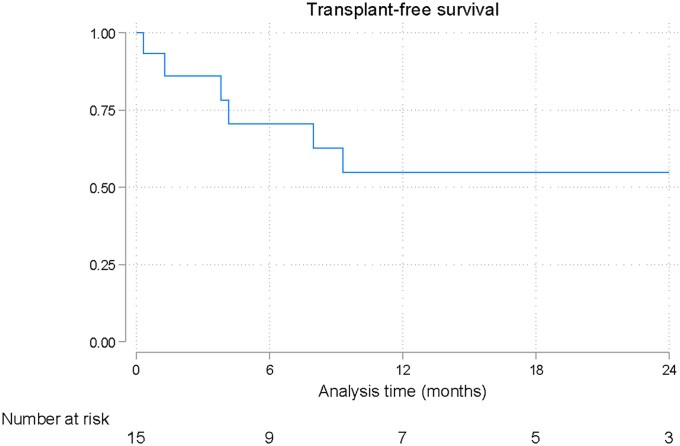
Kaplan-Meier curve for the time to transplantation in paediatric patients with left ventricular assist devices

**Figure 2:**
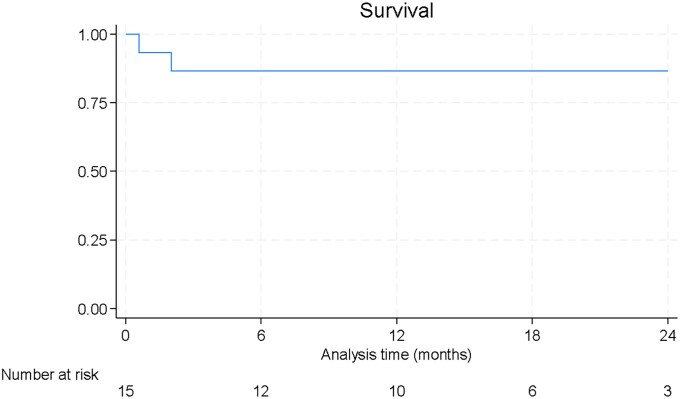
Kaplan-Meier survival curve for paediatric patients treated with left ventricular assist devices

## Methods

### Design and patients

This retrospective cohort study involved 15 paediatric patients with end-stage heart failure who were treated with the HM3 device. The patients were managed at a single tertiary referral cardiac centre between September 2022 and June 2024. Patients over 14 years were excluded (adult age per Saudi definition) and those with VADs other than HM3 were excluded from the study.

### Data and outcomes

The baseline data included age, sex, weight, BSA, blood group, right ventricular function (classified as normal, mild, moderate, or severe depression), left ventricular end-diastolic diameter (LVEDD), previous cardiac surgeries, anticoagulation status, preoperative extracorporeal membrane oxygenation, and preoperative diagnosis. The severity of heart failure was assessed via the Interagency Registry for Mechanically Assisted Circulatory Support (INTERMACS) score.^16^ Operative data included any associated surgeries and the duration of cardiopulmonary bypass (CPB).

Postoperative outcomes included the length of stay in the intensive care unit (ICU) and hospital, as well as the incidence of stroke, bleeding requiring re-exploration, gastrointestinal bleeding, dialysis, driveline infections, thrombosis, and right ventricular function. The long-term outcomes included survival rate and time to heart transplantation.

#### Surgical technique

All patients underwent HM3 LVAD (left ventricle assist device) implantation via median sternotomy under CPB using standard aortic and right atrial cannulation. Procedures were performed on a beating heart at normothermia, except in one patient with a large left ventricular thrombus who required brief aortic cross-clamping and cold blood cardioplegia for clot evacuation.

In one case of congenitally corrected transposition of the great arteries (cc-TGA), pulmonary artery band (PAB) removal and right ventricular outflow tract (RVOT) augmentation were also performed before LVAD placement.

All patients were successfully weaned from CPB on inotropic support with stable hemodynamics and satisfactory device performance. No intraoperative mortality or device-related complications occurred

#### Ethical considerations

This study was conducted in accordance with the ethical principles outlined in the Declaration of Helsinki,^17^ the ICH Harmonized Tripartite Good Clinical Practice Guidelines,^18^ the policies and guidelines of the Research Advisory Council of the King Faisal Specialist Hospital & Research Centre, and the laws of Saudi Arabia. The local Institutional Review Board approved the study, and the requirement for patient consent was waived.

### Statistical analysis

Continuous data are presented as the means and standard deviations if normally distributed and as medians (Q1–Q3) if non-normally distributed. Categorical data are presented as numbers and percentages. Time-to-event data were plotted via Kaplan-Meier curves.

## Results

### Baseline characteristics

Fifteen patients received the HM3 device. The mean age was 11.07 ± 1.67 years, with 66.67% being female. One patient had a previous patent ductus arteriosus ligation. The most common diagnosis was dilated cardiomyopathy (*n* = 14), and the LVEDD was 6.9 ± 1.17 cm. Additionally, one patient had a left ventricular clot, and another had cc-TGA. The most common INTERMACS score was 3 (*n* = 7) (*Table 1*).

**Table 1:** Baseline data of the paediatric patients who received heartmate 3

Variables	(*n* = 15)
**Age (years) (mean ± SD)**	11.07 ± 1.67
**Female [*n* (%)]**	10 (66.67%)
**Weight (kg) (mean ± SD)**	37.23 ± 14.02
**Body surface area (m^2^) (mean ± SD)**	1.2 ± 0.26
**Blood group [*n* (%)]**	
A+	1 (6.67%)
AB+	2 (13.33%)
B+	1 (6.67%)
O+	11 (73.33%)
**Right ventricular function [*n* (%)]**	
Normal	7 (46.67%)
Mild depression	1 (6.67%)
Moderate depression	4 (26.67%)
Severe depression	3 (20%)
**Left ventricular end-diastolic diameter (cm) [mean ± SD]**	6.9 ± 1.17
**Previous cardiac surgery [*n* (%)]**	1 (6.67%)
**Preoperative heparin [*n* (%)]**	1 (6.67%)
**Preoperative ECMO**	0
**Preoperative diagnosis [*n* (%)]**	
Dilated cardiomyopathy	13 (86.67%)
Dilated cardiomyopathy + left ventricular clot	1 (6.67%)
Dilated cardiomyopathy + cc-TGA	1 (6.67%)
**INTERMACS score [*n* (%)]**	
1	2 (13.33%)
2	5 (33.33%)
3	7 (46.67%)
4	1 (6.67%)

ECMO, extracorporeal membrane oxygenation; cc-TGA, congenitally corrected transposition of the great arteries.

All the patients had LVEDD > 5.5 cm, except two patients. Patient 11’s LVEDD was 5.3 cm and she had a BSA of 1.35 m^2^. She had no postoperative complications after LVAD implantation. She had her heart transplant 239 days after HM3 device implantation. Patient 15’s LVEDD was 5 cm and she had a BSA of 0.85 m^2^ . She had a postoperative stroke after LVAD implantation, but recovered. She had her heart transplant 125 days after HM3 device implantation.

### Surgical procedure

All patients underwent implantation of the HM3 left ventricular assist device via median sternotomy and CPB with mild hypothermia (34°C). One patient had concomitant PAB removal and RVOT augmentation. The median CPB time was 132 min (range: 103–153) (*Table 2*). One patient required an aortic cross-clamp period to remove left ventricular clot (85 min).

**Table 2:** Operative details of the paediatric patients who received a heartmate 3

Variables	(*n* = 15)
**Surgery [*n* (%)]**	
**Heart mate 3**	14 (93.33%)
**Heart mate3 + PAB removal + RVOT augmentation**	1 (6.67%)
**Left ventricular assisted device [*n* (%)]**	15 (100%)
**Cardiopulmonary bypass time (min) [median (Q1–Q3)]**	132 (103–153)

PAB, pulmonary artery banding; RVOT, right ventricular outflow tract.

### Postoperative outcomes

The median ICU length of stay was 42 days (range: 24–58); the median hospital length of stay was 56 days (range: 37–68). Postoperative complications included stroke in two patients, bleeding requiring re-exploration in one patient, pneumonia in two patients, driveline infection in two patient, and device thrombosis in one patient (*Table 3*).

**Table 3:** Postoperative outcomes of paediatric patients who received a HeartMate 3

Variables	(*n* = 15)
**Length of ICU stay (days) [median (Q1–Q3)]**	42 (24–58)
**Length of hospital stay (days) [median (Q1–Q3)]**	56 (37–68)
**Stroke [*n* (%)]**	2 (13.33%)
**Bleeding requiring re-exploration [*n* (%)]**	1 (6.67%)
**Gastrointestinal bleeding [*n* (%)]**	1 (6.67%)
**Other bleeding (pulmonary haemorrhage) [n (%)]**	1 (6.67%)
**Dialysis [*n* (%)]**	0
**Pneumonia [*n* (%)]**	2 (13.33%)
**ALT (units) (mean ± SD)**	25.63 ± 12.80
**AST (units) [median (Q1–Q3)]**	29 (19–48)
**Driveline infection [*n* (%)]**	2 (13.33%)
**Device thrombosis [*n* (%)]**	1 (6.67%)
**Hospital mortality [*n* (%)]**	2 (13.33%)
**Discharge right ventricular function [*n* (%)] (*n* = 11)**	
Normal	2 (18.18%)
Mildly reduced	4 (36.36%)
Moderately reduced	3 (27.27%)
Severely reduced	2 (18.18%)
**Postoperative anticoagulation [*n* (%)]**	
Warfarin + aspirin	15 (100%)

#### Mortality

In-hospital mortality was reported in two patients. One patient had an ischaemic stroke and fully recovered. Unfortunately, he had haemorrhagic transformation 2 weeks later with mass effect and tonsillar herniation. This patient had an INTERMACS class of 2, a LVEDD of 6.7 cm, and moderately depressed right ventricular function. The second patient experienced device thrombosis 18 days after her surgery. She had a preoperative INTERMACS class of 1, normal right ventricular function, and an LVEDD of 9.2 cm.

#### Stroke

Of the two patients who developed postoperative stroke, one had an ischaemic stroke that had had haemorrhagic transformation, leading to brain herniation and death. The other patient had full recovery and was transplanted at 125 days.

### Follow-up

The median follow-up time was 17 months (Q1–Q3: 2–22 months). Nine patients (60%) underwent transplantation, with the median time to transplant being 9 months (Q1–Q3: 2–21) (*Figure 1*). The transplantation-free survival rate at 1 year was 55%. No mortality was reported beyond hospital discharge, and the survival rate at 1 year was 87% (*Figure 2*).

## Discussion

### Study summary

This retrospective cohort study investigated the short-term outcomes of HM3 VAD in 15 paediatric patients with end-stage heart failure. The median age was 11.07 years, the most common diagnosis was dilated cardiomyopathy, and the most common INTERMACS score was 3. The median CPB time was 132 min. The median ICU stay was 42 days, and the hospital stay was 56 days. Stroke occurred in two patients, driveline infection in one patient and mortality in two patients. Transplantation-free survival at 1 year was 55%, and no mortality was reported beyond hospital discharge.

### Comparison with the literature

Left VADs are emerging as options for managing end-stage heart failure in paediatric patients because of the limitations of heart transplantation in this age group, including the limited number of donors and the size match.^19,20^ Initial VADs were designed to adapt to the needs of adult heart failure patients, and EXCOR (Berlin Heart GmbH, Berlin, Germany) was the first VAD implanted in children.^21^ This was followed by several other types of VADs, including HeartWare (HVAD, Framingham, MA, USA) and infant Jarvik 2015 (Jarvik Heart, Inc., New York, USA).^19^

HeartMate 3, a continuous flow device, was approved for use in paediatric patients in 2020 by the Food and Drug Administration.^22^ VADs are used in children as a bridge to transplantation, and several studies have reported that VADs are a successful bridge to transplantation in heart failure patients regardless of heart failure aetiology.^23,24,25^ HM3 showed promise in paediatric patients. O’Connor *et al*.^13^ evaluated the outcomes of HM3 in 35 patients; the median age was 16 years, and the most common diagnosis was dilated cardiomyopathy. The median follow-up was 78 days, and they reported one case of mortality from the device and a 57% transplantation rate. A striking difference between our series and others is the age and BSA of the patients, since our cohort included younger patients. The younger patients included in our study could explain the differences in outcomes between our study and others.

Stroke is a significant complication in patients on VADs. Niebler *et al*.^26^ studied 662 patients from the Pediatric Interagency Registry for Mechanical Circulatory Support and reported cerebrovascular accidents in 87 patients (11%). Dogan *et al*.^27^ reported zero strokes in 11 children who received HM3. In our cohort, stroke was reported in two patients. Several risk factors could contribute to stroke in this subset of patients. A study demonstrated a higher stroke rate in pulsatile flow than in continuous flow VADs; additionally, the stroke rate was greater in high-risk patients and in centres with small volumes.^26^ Sinclair and associates reported a higher stroke rate in patients with a single ventricle and cardiomyopathy.^28^

Anticoagulation management after VAD implantation is a significant clinical issue and is needed to prevent device thrombosis and stroke. Compared with aspirin and warfarin, aspirin is an effective anticoagulation regimen for adult patients.^29^ However, the optimal anticoagulation strategy in children is still controversial. In this study, all patients were bridged to postoperative aspirin and warfarin via heparin infusion. Despite the promising results of the ARIES trial, we chose to use aspirin in our patient cohort for two reasons.^30^ First, there is a high prevalence of consanguinity in our population, with increased incidences of hypercoagulable states and hematological disorders like sick cell disease.^31,32^ Second, compared with adults those population the device works with low flow states, which can result in thrombosis risk

Driveline infection is the most common type of VAD-associated infection and has a substantial negative effect on patients’ health and outcomes.^33,34^ The risk of infection increases with the duration of VADs; it is reported to be 9% in the first 3 months, and the peak of infection is reported to be reached after 6 months.^34,35^ Our study reported a diffuse infection in two patients. Several risk factors could contribute to the development of infection, including patient comorbidities, driveline material and centre experience.^26,33,34^

### Implications

The results suggest that the HM3 device can be a viable option for paediatric patients with end-stage heart failure, with acceptable survival rates and a low incidence of adverse events compared with international registry outcomes.^36^ These findings highlight the need for further studies to establish long-term outcomes and optimal management strategies for this vulnerable population. The relatively high transplantation rate within the follow-up period underscores the potential for HM3s to act as an effective bridge to transplantation.

## Limitations

This study has several limitations, including its small sample size, which limits the generalizability of the findings. The study was conducted at one centre, which may not reflect practices or outcomes at other facilities. The median follow-up of 17 months may not capture long-term complications or survival trends. The observational design may introduce biases and confounding variables that could affect reported outcomes.

## Conclusion

The HM3 device shows promising short-term outcomes in Saudi paediatric patients with end-stage heart failure, demonstrating satisfactory survival and manageable complication rates. While these results are encouraging, they should be interpreted cautiously given the limited sample size and early experience. To solidify these findings, it is crucial to pursue multicenter collaborations and longer follow-up studies. This approach will not only confirm safety and improve patient selection but also significantly strengthen paediatric VAD programmes in developing regions.

## Declarations

### Data Availability

The Authors confirm that the data supporting the findings of the this study are available within the article and its supplementary.

### Pre-registered Clinical Trial Number

None supplied.
